# Osteomyelitis Caused by Citrobacter koseri in a Young Immunocompetent Man

**DOI:** 10.7759/cureus.62627

**Published:** 2024-06-18

**Authors:** Samrawit W Zinabu, Yashmith Duddukunta, Swathi Muttana, Jimmy Smith, Bharadwaj Adithya Sateesh, Miriam B Michael

**Affiliations:** 1 Internal Medicine, Howard University Hospital, Washington, DC, USA; 2 Internal Medicine, University of South Florida, Tampa, USA; 3 Family Medicine, Geisinger Health System, Lewistown, USA; 4 Orthopedics, Howard University Hospital, Washington, DC, USA; 5 Medicine, University of Maryland Midtown Campus, Baltimore, USA; 6 Medicine, American University of Antigua, Osbourn, ATG; 7 Internal Medicine, University of Maryland, Baltimore, USA

**Keywords:** immunocompromised, musculoskeletal, citrobacter koseri, discitis

## Abstract

*Citrobacter koseri* is a non-sporulating, motile, gram-negative, facultative anaerobic bacteria found in various environmental sources, including the human intestine. It is considered an opportunistic infection as it typically causes infection in newborns, elderly, and immunocompromised patients. Common sites of infection are the urinary tract, gastrointestinal system, and respiratory tract in immunocompromised adults, as well as the bloodstream and meninges in newborns. However, osteomyelitis secondary to *C. koseri* is very rare. We present an unusual case of *Citrobacter* osteomyelitis in a healthy young man with no identifiable risk factors.

## Introduction

*Citrobacter koseri* is a non-sporulating, anaerobic, motile, gram-negative bacteria of the *Enterobacteriaceae *family [1]. It is part of the normal flora in mammalian gastrointestinal and urinary tracts and is considered an opportunistic infection [2]. *C. koseri* is well known for its involvement in pediatric central nervous system infections like neonatal brain abscesses and meningitis [3]. In adults, infections typically occur in immunocompromised individuals or within healthcare settings [4]. Common sites of infection are the urinary tract, GI, and CNS, but osteomyelitis secondary to *Citrobacter *bacilli is rare [4]. If osteomyelitis develops, the sources are hematological spread or are caused by direct trauma or surgical procedures. Our patient's primary source of *C. koseri* remains unclear since he did not have previous hospitalization, invasive procedures, or any associated risk factors or immunodeficiency. The treatment principle for *C. koseri* is the same as that of other *Enterobacteriaceae *families [5]. The most common approach has been to use multidrug therapy, typically involving a penicillin or cephalosporin antibiotic combined with an aminoglycoside [5].

## Case presentation

An 18-year-old African American male with no known past medical history and up-to-date immunizations presented to the University of Maryland Hospital initially with back pain radiating to his legs and tingling in his feet. The pain started after an injury he received working as a mover. He lifted something heavy and heard a "pop." He then proceeded to have worsening back pain over the next week and presented to the emergency department for evaluation. Previous workup of his back pain included evaluation by primary care physician, podiatry, urgent care, and orthopedics outpatient. At an emergency visit a month ago, he was given acetaminophen and lidocaine patches. At an outpatient visit to podiatry, he was also given a course of steroids with no improvement in his symptoms. He was started on cyclobenzaprine two weeks before his presentation when he presented again to the emergency with difficulty walking.

**Figure 1:**
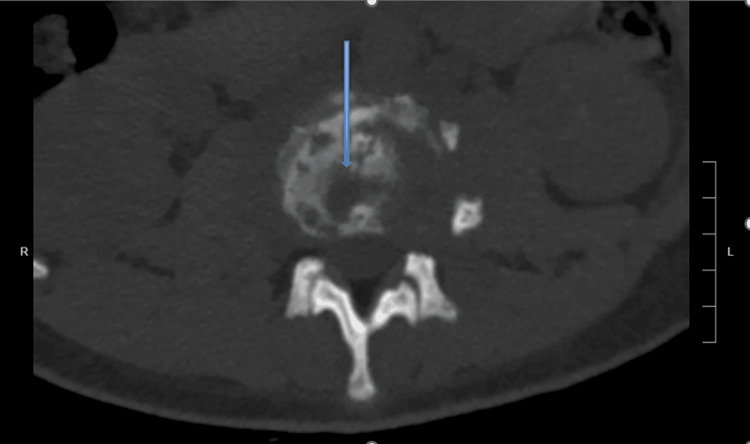
CT of the lumbar spine without contrast showing extensive erosive bony changes at the L2-L3 levels, with loss of vertebral body height consistent with known discitis osteomyelitis. There is severe left and mild to moderate right neural foraminal narrowing at the L2-L3 levels. CT: computed tomography

A week later, he was eventually seen and investigated by orthopedics. However, the results were nonrevealing. He later developed fever and chills, with a temperature of 38.9°C. A CT scan (Figure 1) of the lumbar spine at that time revealed discitis osteomyelitis.

Two days later, the culture of bone biopsy grew pan-susceptible *C. koseri*. The patient was subsequently admitted and completed one week of IV cefazolin and transitioned to PO cephalexin 500 BID upon discharge due to clinical improvement and down-trending of his C-reactive protein (CRP). The 500 mg BID dose was noted to be low for bone penetration and was subsequently increased to 1 g q6h at his follow-up appointment with the pediatric department a month later. At a second follow-up six weeks after discharge, the patient complained of worsening back pain and new onset left flank pain and was sent to the emergency unit for further evaluation.

**Figure 2:**
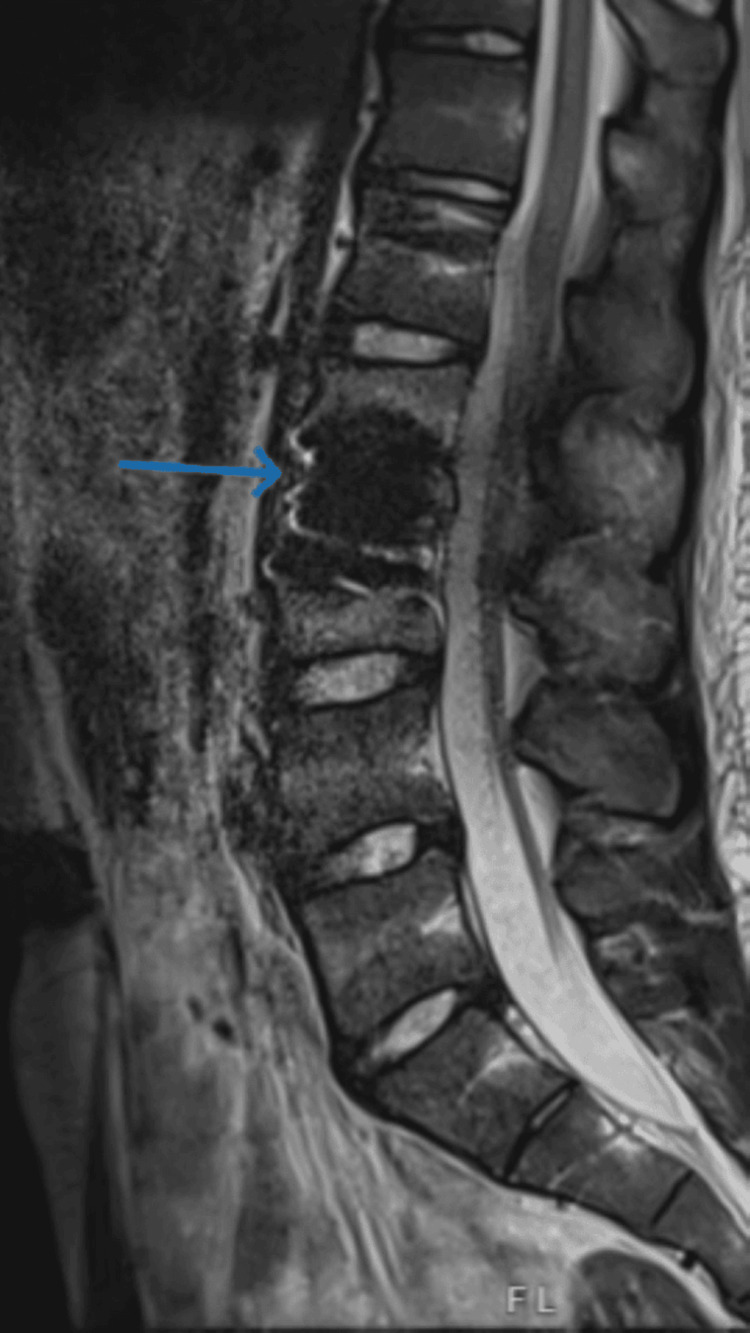
MRI of the lumbar spine with and without contrast showing worsening discitis osteomyelitis involving the entire L2-L3 vertebral bodies and intervertebral disc, with significant loss of disc height and an extension of infection and enhancement into bilateral psoas muscle without abscess. MRI: magnetic resonance imaging

During the emergency unit visit, the physical examination noted a grossly decreased range of motion in the back secondary to pain and tenderness to palpation over L2/L3 and paraspinal muscles. Lower extremities exhibited intact sensation without a reduced range of motion. He also denied bowel and bladder incontinence. Labs were negative for leukocytosis and a stable erythrocyte sedimentation rate (ESR), with an up-trending CRP from 17 to 23. Pediatric infectious disease was consulted, and the MRI (Figure 2) showed worsening osteomyelitis and discitis of the L2/L3 vertebrae, moderate to severe stenosis at the same level, and inflammation of the bilateral psoas muscles without evidence of abscess.

Pediatric infectious disease recommended that the patient be admitted again, with IV cefazolin 2 g every eight hours. His BMI was 19, and the HIV test was non-reactive. He underwent an immunology evaluation, which included tests for IgG, IgA, and IgM, antibody titers for *Haemophilus influenzae* type B, tetanus, and 14 serotypes of pneumococcus to assess antibody function, as well as CH5 and a lymphocyte panel via flow cytometry. The lymphocyte panel included CD3, CD4, CD8, CD19, and CD16/56 markers, all yielding results within the normal range. During his hospital stay, debridement and hardware placement in the lumbar spine were done. After three weeks, there was a notable clinical improvement, and subsequent MRI scans showed the absence of residual infection.

The patient has no history of recurrent, severe, or unusual infections and no history of other joint pain, swelling, or trauma. He did work at a moving company and sometimes lifted large/heavy items, but he has not had any recent injuries. Family history is notable for the dad, who has sickle cell trait and no known family history of immunodeficiency or autoimmune disease. The patient does not have a diagnosis of sickle cell trait or disease. He takes no medications and has never had surgery.

## Discussion

*Citrobacter *species in nature are frequently found in water, soil, and food. In mammals, the most common isolate is *C. koseri*, formerly known as *Citrobacter diversus* [6]. It is usually found in the gastrointestinal tract. *Citrobacter *is a gram-negative bacillus, which is oxidase-negative and utilizes citrate as the sole carbon source [6]. *Citrobacter *infection is rare and responsible for less than 1% of all gram-negative infections, with over 70% being nosocomial and associated with invasive procedures like catheterization and genitourinary instrumentation [7]. *Citrobacter *is considered a low-virulence organism, and the risk factors for infection are not particularly known. They most commonly infect the urinary tract, gastrointestinal tract, wound, bloodstream, and central nervous system of immunocompromised adults [7]. *C. koseri* also causes meningitis and brain abscesses in neonates.

*C. koseri* is rarely involved in infections of the musculoskeletal system. Only 14 musculoskeletal system infections have been reported since the 1980s [8]. Studies of these infections have observed that it is more common in the elderly, neonates, and immunocompromised groups, with diabetes mellitus and peripheral vascular disease being a particular risk for the development of pedal osteomyelitis [8]. Infections are frequently hospital-acquired, and the patients commonly have a history of invasive procedures resulting in significant injury or inflammation at the site of primary infection before bacteremia occurs. Only three cases of spondylodiscitis by *Citrobacter *were reported in the literature [9]. All were caused by *C. koseri* in elderly immunocompromised individuals above 70 years of age with multiple comorbidities. This case is among a few reported cases of *C. koseri* osteomyelitis in immunocompetent individuals.

The gold standard diagnostic method for *Citrobacter *osteomyelitis is bone biopsy and culture. MRI is the preferred method for diagnosing spinal osteomyelitis. There is very little literature about treating osteomyelitis in *Citrobacter *since most literature mentions the treatment of meningitis or sepsis in infants. *C. koseri *is naturally resistant to aminopenicillins and carboxypenicillins [10]. Many antibiotics, including aminoglycosides, carbapenems, cephalosporins, chloramphenicol, and quinolones, are used for the treatment of *C. koseri* infections [10]. Of concern is that *C. koseri* gains antibiotic resistance over time against various types of antibiotics through chromosomal and plasmid-mediated determinant genes [11]. This makes having a standard choice challenging because there is a sustained increase in anti-bacterial resistance, forcing any antimicrobial therapy to be based on an anti-bacterial susceptibility pattern [11]. Colistin and tigecycline are reserved for multidrug-resistant isolates [12]. The total duration of treatment varies between four and 16 weeks, depending on comorbidities, isolated microorganisms, and the presence of neurological complications [12].

## Conclusions

We report a rare case of *C. koseri* osteomyelitis in a healthy young man. This case emphasizes the importance of maintaining a high index of suspicion and broadening the differential diagnosis for osteomyelitis, even in patients without established risk factors. Additionally, it spotlights carefully selecting antibiotics for *Citrobacter* infection to prevent long-term complications.
